# Dr. Kentish on Dispensaries of Heat

**Published:** 1810-11

**Authors:** E. Kentish

**Affiliations:** Bristol


*To the Editors of the Medical and Physical Journal.*

GENTLEMEN,

FROM some observations in the historical sketch of the progress of medicine in the year 1809, by Mr. Royston, I am happy to find the attention of the faculty called to appreciate the the effects of baths. So much has been written for some years past on the subject of applying modified temperature to the body, as a curative means in disease; that it is an object of the greatest importance, that the faculty in every town in the kingdom, should have it in their power to avail themselves of such aid. The object I have in view, is to establish Dispensaries of heat, where the faculty of the place may order a dose of heat to be administered, with the same facility and exactness, as they now do, with every other article in the shop of the apothecary. I have lately superintended the fitting up of a Dispensary of this kind, which possesses seven different modes of applying heat, in a small room of twelve feet by eighteen: namely, a cold bath, a warm bath, a vapour bath, a cold shower bath, a warm shower bath, a cold douche (or forcible stream of water directed to a particular part) and a warm douche. As the execution of a plan of this nature requires the united assistance of a variety of artists; perfection cannot be expected, without having sustained a variety of failures. At a future period, I purpose to give a description of the manner of constructing these Dispensaries; at present my establishment is open to the inspection of the faculty, and I shall have much pleasure in giving assistance to form similar establishments. For the success of such an institution as I propose, it would require the joint support of the whole faculty of the place in which it may be situated.

To obtain this apparently impossible object, I know of no means so likely to effect the purpose, as to make it the interest of the whole to give it their support; probably some scheme like the following would succeed. Suppose it would require a thousand pounds to fit up a Dispensary of this class, divide the capital into a hundred shares, which should be taken by the *whole* of the faculty of the place, in such proportions as they may agree upon; every individual only to have one vote, however numerous might be his shares. The business of the establishment should be conducted by a committee of three members, to be chosen annually, to whom the superintendant should be answerable for his conduct. Much will depend on the intelligence of this servant; for, as the practice of vapour bathing advances, many substances will be found to exert a powerful influence on the system, when introduced by the medium of the lungs; for instance, in cases of spasmodic asthma, I have found much benefit by making the vapour pass through a sponge, on which a dose of compound spirit of æther, and tincture of opium, was placed. In an appendix to my Essay on Baths, I have given cases in which the good effects of an anodyne vapour, is sufficiently apparent in catarrh, and bronchitis. I shall for the

 present present forbear alluding to the extensive range of usefulness in medicine, which we may be led to by this fact. Should these hints induce the co-operation of any set of medical men, in the investigation of a subject so important to the best interests, both of society and of the faculty, they may be assured of my aid and assistance.

I am, Gentlemen, yours, &c.

E. KENTISH, M. D.*

* The Editors feel much indebted to Dr. Kentish for his liberal observations on the application of modified temperature to animal bodies. On this subject, which is undoubtedly of considerable importance as a curative process, they hope to be the medium for conveying information to the medical public; and as Dr. Kentish is devoting a particular attention to it, they have a reasonable expectation that he will occasionally favour them with remarks on the mode of application, and on the facts that may result from such application. The Hon. Basil Cockrane is still pursuing, with ardor, his experiments with the Vapour Bath; and itis said, greatly improving his apparatus. As this gentleman proceeds in his investigation, the Medical and Physical Journal will not be inattentive to the facts that may arise.

